# Methods of Assessment and Clinical Relevance of QT Dynamics

**Published:** 2005-07-01

**Authors:** Beata Sredniawa, Agata Musialik-Lydka, Piotr Jarski, Anna Sliwinska, Zbigniew Kalarus

**Affiliations:** First Department of Cardiology, Silesian Medical School, Silesian Center for Heart Diseases, Zabrze, Poland

**Keywords:** QT interval, ventricular repolarisation, QT dynamics, arrhythmic risk, QT interval prolongation

## Abstract

The dependence on heart rate of the QT interval has been investigated for many years and several mathematical formulae have been proposed to describe the QT interval/heart rate (or QT interval/RR interval) relationship. While the most popular is Bazett’s formula, it overcorrects the QT interval at high heart rates and under-corrects it at slow heart rates. This formulae and many others similar ones, do not accurately describe the natural behaviour of the QT interval. The QT interval/RR interval relationship is generally described as QT dynamics. In recent years, several methods of its assessment have been proposed, the most popular of which is linear regression. An increased steepness of the linear QT/RR slope correlates with the risk of arrhythmic death following myocardial infarction. It has also been demonstrated that the QT interval adapts to heart rate changes with a delay (QT hysteresis) and that QT dynamics parameters vary over time. New methods of QT dynamics assessment that take into account these phenomena have been proposed. Using these methods, changes in QT dynamics have been observed in patients with advanced heart failure, and during morning hours in patients with ischemic heart disease and history of cardiac arrest. The assessment of QT dynamics is a new and promising tool for identifying patients at increased risk of arrhythmic events and for studying the effect of drugs on ventricular repolarisation.

## Introduction

The duration of ventricular repolarisation, represented by the QT interval on the standard 12-lead electrocardiogram (ECG), is influenced by a variety of physiological and pathological factors, such as heart rate, autonomic tone, hormone concentrations, drug therapy, presence of heart disease or ventricular dysfunction, electrolyte disturbances, etc. [1], the most important of which is the heart rate. The influence of the autonomic tone on the QT interval has been demonstrated by variations in QT interval duration during fixed rate pacing and by differences between QT intervals recorded during the day and during the night at identical heart rates [2]. The relationship between QT interval and heart rate has been investigated for over 100 years. Many mathematical formulae have been proposed to describe this relationship and to derive a “heart rate-corrected” (i.e. presumably heart rate-independent) QT (QTc) interval, which would allow comparisons between QT intervals recorded at different heart rates. The following methods have been proposed to evaluate the QT interval heart rate (or QT interval/RR interval) relationship: standard heart rate correction formulae applied to recordings acquired under steady-state conditions and non-standard methods, which can be applied to recordings taken under non-steady-state conditions [3]. The latter describe the general relationship between QT interval and RR interval, which is usually described as QT dynamics.

## Standard, steady-state formulae to assess the dependence of the QT interval on the RR interval

These formulae have been derived mainly from resting ECGs. Exponential, logarithmic and linear formulae have been proposed. The two earliest exponential formulae were published in 1920 by Bazett [4], who postulated that the QT interval (in seconds) varies with the square root of the RR interval (in seconds) according to the following equation:


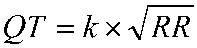


and by Fridericia [5], who proposed a cube-root formula:


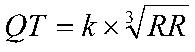


Bazett found that a k value of 0.37 in men and 0.40 in women corresponded to a QT interval measured at a heart rate of 60 beats per minute. The latter has been termed the “heart rate-corrected QT (QTc) interval”. At present, Bazett’s formula is used in the form:


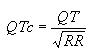


where QT and RR are expressed in seconds. All other formulae for heart rate-correction of the QT interval have also been developed in order to determine whether the QT interval is prolonged in comparison to its predicted value at a reference heart rate of 60 beats per minute (i.e. a RR interval of 1.0 second).

Bazett’s formula has been shown to over-correct (i.e. to yield erroneously short QTc intervals) at slow heart rates and to under-correct (i.e. to erroneously prolong the QTc interval) at high heart rates. Therefore Bazett’s formula can only be applied to correct the QT interval within a range of heart rate between 50 and 90 beats per minute [6]. The same limitation, although to a lesser degree, applies to Fridericia’s formula [1,6].

The most popular logarithmic formula has been proposed by Ashman [7]:

**QT = K1 x log(10 x [RR + K2])**

where K2 = 0.07, and K1 = 0.380 for adult men and 0.390 for adult women. At low heart rates, the values yielded by this formula are too low.

The first linear formula has been proposed by Adams [8]:

for women: **QT = 0.1259 x RR + 0.2789**

for men: **QT = 0.1536 x RR + 0.2462**

Schlamowitz [9] proposed the following formula for the general population: **QT = 0.205 x RR + 0.167**

This formula underestimates the QT interval, yields QTc values that are too high at very high heart rates, although it generally yields values similar to the ones calculated with Fridericia’s formula [1].

Since 1920, dozens of similar formulae have been proposed in addition to the above-described ones, such as the formulae of Mayeda, Sagie, Hodges, Sarma and many others [10]. Different formulae applied to the same QT/RR (or QT/heart rate) data can render QTc intervals that vary by several milliseconds. The purpose of heart rate correction is to receive QTc value independent of the underlying heart rate.

In order to assess the performance of a particular heart rate-correction formula, the correlation QTc intervals calculated using the formula and the RR intervals can be assessed. If it differs from zero, the correction formula is not truly successful, which has been demonstrated with some of the above described formulae [10]. The application of all these formulae are limited to data acquired under steady-state conditions. Therefore they do not describe the biological process of adaptation of repolarisation to abrupt changes of heart rate, i.e. under non-steady-state conditions [3]. The so-called “bin” method has been proposed as an alternative to the use of heart rate correction formulae. The idea is to compare QT interval recorded at identical or very similar heart rates (e.g. preceded by the same RR intervals), for example, before and after drug treatment, some intervention, etc. Pairs of RR and QT intervals are collected and distributed into “bins” according to the immediately preceding RR value. Another pairs, obtained after drug treatment, are collected at the same RR but with new QT and are compared to the baseline pairs. The advantage of the “bin” method is the ability to compare QT interval duration for any of RR interval. It is particularly important in infants, in patients with slow or high rates and when studying drugs which induce significant changes in heart rate [11,12]. An obvious limitation of the “bin” method is the fact that sufficient number of QT intervals recorded at identical heart rates are not always available for comparison, e.g. when a drug has changed substantially the heart rate. Another limitation of the “bin” method stems from QT hysteresis, e.g. the QT interval duration depends not only on the immediately preceding RR interval. Moreover, due to autonomic tone the QT interval duration can be different at identical heart rates. It has been demonstrated during constant pacing rates [2,13]. Hence this is another limitation of the “bin” method.

## Non-steady state methods of assessment of the QT interval/RR interval relationship

These methods use continuously recorded ECG data. Ambulatory ECG (Holter) monitoring is a useful tool, as it allows the assessment of QT dynamics under different physiological conditions, such as physical activity and rest [14]. All techniques require high quality recordings with few artefacts, a high signal-to-noise ratio and a stable isoelectric baseline and great care in abnormal T wave morphology, especially biphasic [15]. It is particularly important to use an appropriate method for the detection of the end of the T wave. Several methods have been proposed for the detection of the T wave offset. The *threshold methods* define T offset as an intercept of T wave or of its derivative with a threshold above the isoelectric line. The *slope methods* localize T offset as an intercept between the slope of descending part of T wave and isoelectric line, or a threshold line above it. The *tangent method* determines T offset as a tangent to the steepest point of the descending limb of T wave or with a line trough the T wave peak and the maximum slope point [16]. Since the methods based on Holter recordings describe the natural adaptation of the QT interval to changes in heart rate, it is important to use software which performs “beat-to-beat” analysis [3,14,17,18]. Both linear and non-linear methods of assessment of QT dynamics have been described.

## Linear assessment of QT dynamics and its clinical relevance

This method computes linear regressions between QT and RR intervals and measures the regression slope. The slope defines the percentage of QT interval change per unit of RR interval change. A steep QT/RR slope indicates greater changes of the QT interval with changes in heart rate, greater QT prolongation at slow heart rates, and appropriate or even increased QT shortening at fast heart rates. A flat slope indicates less rate-dependence, or even a lack of QT interval shortening at fast heart rates [15]. Different methods have been used to measure the slope. Some investigators use pooled data, while others measure the slope from finite periods, e.g. for time intervals chosen from the entire 24-h recording, or from RR intervals between 700 and 1000 ms [18-21]. In healthy individuals, the slope exhibits circadian variations - it is steeper during the day than during the night, with peak values in the early morning hours. The value of the slope depends on whether it is measured from pooled data or from narrow periods. The reported slopes from pool data are 0.17 for 24 h, 0.13 for daytime, and 0.09 for night time, whilst the corresponding measurements from narrow periods are 0.13 - 0.14, 0.08, and 0.06, respectively [18][19][21]. The slope measured from narrow periods is usually flatter than that estimated from pooled data. Shortening of the sampling periods results in loss of information about the shortening of the QT interval at fast heart rates, which causes flattening of the slope. In addition, the slope is steeper in women than in men [18].

It has been documented that QT/RR relationship differs substantially among healthy subjects [22]. The inter-individual variability has been shown to be greater than the intra-individual variability. The individual QT/RR pattern assessed under similar conditions is stable, “fingerprint-like” in each subject and differs substantially between different subjects which suggests that the inherent, probably genetically determined differences in cardiac repolarization are greater than the enviromental or autonomic differences. On the basis of these observations has been suggested that in investigations that require precise measurement of the QTc interval, such as drug safety studies, the individual optimum QT/RR regression model should firstly be assessed in drug free-state in order to derive individual QT correction formulae [22][23]. Most recently it has been observed that the individual QT/RR relationship still exhibits residual variability related to autonomic changes [12]. These researchers suggested that the evaluation of the individual QT/RR relationship is appropriate only during stability of autonomic nervous system influences for each study period [12].

**Figure 1:**
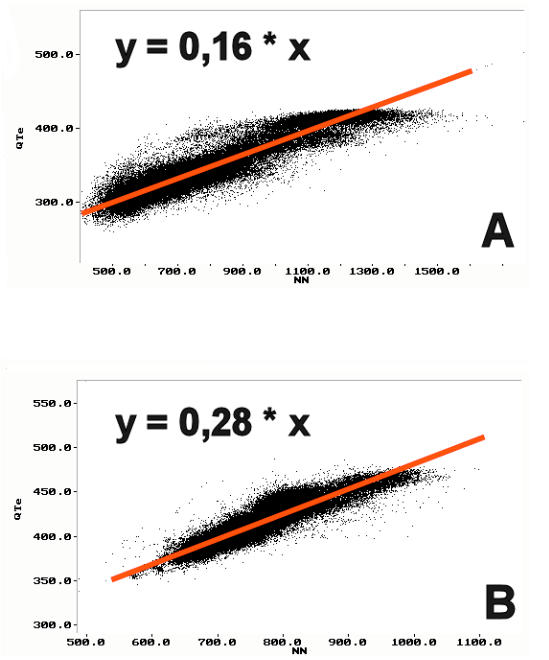
QT/RR linear regression slopes recorded from a healthy subject (A), and from a patient with ischemic heart disease and in NYHA functional class IV (B).

Steeper QT/RR linear regression slopes have been reported in many diseases as well as in patients at risk of sudden cardiac death.[15] Representative examples of 24-h slopes recorded in a healthy subject and in a patient with advanced ischemic heart disease are presented in Figure 1.

Steep slopes have been recorded in survivors of myocardial infarction who later died suddenly [24]. Recently this has been confirmed by the results of the QT dynamics evaluation of patients enrolled in the European Myocardial Infarct Amiodarone Trial (EMIAT) who suffered arrhythmic cardiac deaths (ACD) [25]. In the ACD group, the daytime, night time and morning slopes were 0.25, 0.23 and 0.27, respectively. The analysis of the type of cardiac death: (ACD vs non-ACD) revealed significantly steeper morning slope in ACD group than in non-ACD, while daytime and night time slopes did not differ between both groups [25]. Increased QT dynamics has also been reported to be an independent risk factor in patients with heart failure [26]. Some researchers have found that survivors of acute myocardial infarction who achieved a TIMI flow III in the infarct-related artery after primary percutaneous coronary angioplasty had flatter slopes than patients with TIMI II flow [27]. They concluded that incomplete revascularization altered the QT dynamics and, therefore, may represent a substrate for malignant ventricular tachyarrhythmias. Treatment with beta-aderenergic blockers decreases the QT/RR slope [28]. Carvedilol decreases the QT/RR slope significantly in survivors of acute myocardial infarction, while metoprolol also showed a similar trend [20]. The QT/RR slope has been shown to be related to specific genotypes of the congenital long QT syndrome (LQTS). Patients with LQTS type 1 and type 2 (K^+^ channel abnormalities) have flatter slopes and impaired shortening of the QT interval at fast heart rates, whilst the type 3 LQTS patients is characterised by a steeper slope and QT prolongation at slow heart rates [29].

## Non-linear and non-steady-state assessment of QT dynamics and its clinical relevance

**Figure 2:**
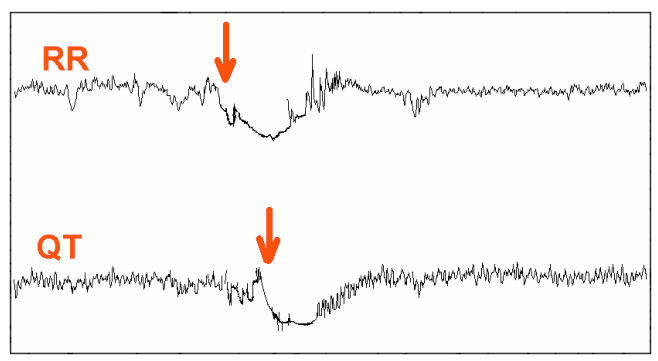
QT lag. Upper tracing: trend of RR intervals. Lower tracing: trend of QT intervals. The red arrows indicate the onset of change in heart rate and the delay in the corresponding change in QT interval.

Most modern advanced Holter systems allow continuous and simultaneous “beat-to- beat” analysis of RR and QT intervals. The QT interval adapts to heart rate changes with a delay known as QT hysteresis or QT lag [30]. When the change in the heart rate persists for several minutes, the QT lag is visible on the trend of QT and RR intervals. Figure 2 is an example of such QT lag during Holter monitoring.

**Figure 3:**
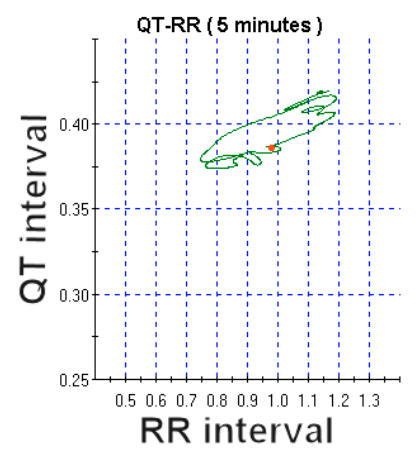
QT interval hysteresis versus RR dynamic relationship.

It takes several minutes for the QT interval to adapt to the new heart rate after an abrupt change. The QT adapts more slowly to decelerations than to accelerations of the heart rate. The plot of QT versus RR intervals during dynamic adaptation of repolarisation to heart rate changes forms a loop known as hysteresis [31]. Figure 3 is an example of QT hysteresis during a 5 minute heart rate change recorded on Holter.

**Figure 4:**
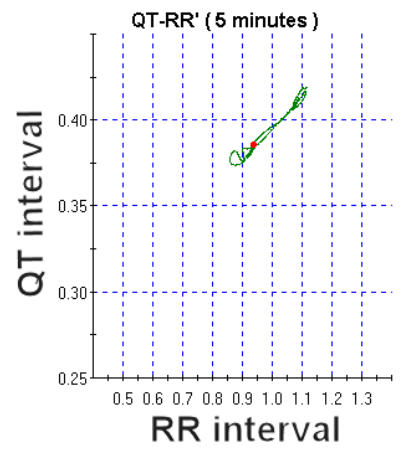
Compensation of QT lag using the same dataset as presented in Figure 3. See text for detailed explanations

Since the heart rate varies over time, the QT/RR relationship is not constant and the QT changes tend to lag behind the heart rate changes. One approach to the evaluation of this natural behaviour of QT dynamics is to evaluate it over several minutes of stable narrow ranges of heart rate. This method, however, causes loss of information regarding QT dynamics over different ranges of heart rate. To overcome this limitation, a new non-linear method of QT dynamics assessment from non-steady-state recordings was developed in 1998 by Neilson [32,33]. The first step is to compensate for the QT lag by resynchronising the variations in QT and RR. This eliminates the loop and replaces it by a compensated curve. Figure 4 is an example of QT/RR data (same as on Figure 3) after resynchronisation.

The next step is a continuous calculation of the QT/RR slope inside 5-min windows, during periods of strong correlation between RR and QT (r >0.8). The results are used for the continuous correction of the QT interval in the equation:

**QT*cj*  = QT (RR) ^- J^**

(all intervals in seconds)

**Figure 5:**
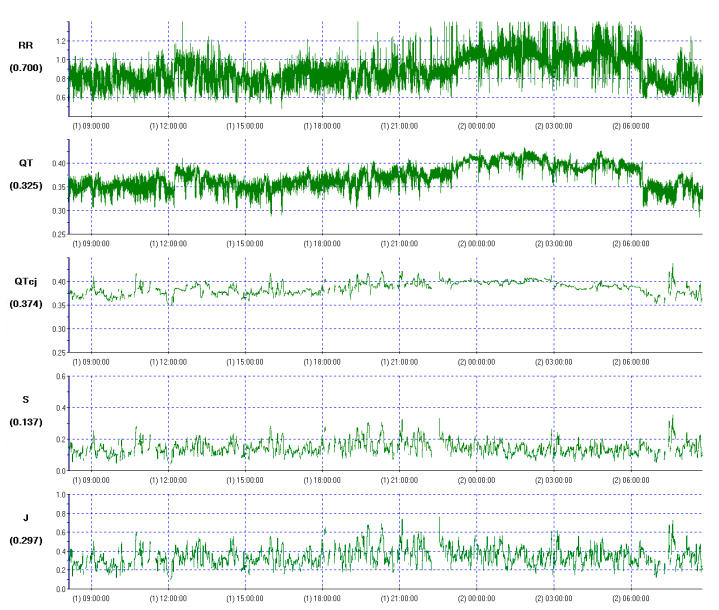
Non-steady-state and non-linear analysis of QT dynamics performed in healthy subject, using Neilson’s method. Mean QTcj = 402 ms, mean J = 0.31.

In this formula, the J exponent, calculated continuously from the equation J=S(RR/QT), varies over time. The equation shows that the J exponent is not constant over the 24-hour period and varies in healthy subjects between 0.28 and 0.38 (mean = 0.32), with a daytime value of 0.25 (i.e. one half of Bazett’s). The QTcj ranges between 384 and 448 ms. This method is highly reproducible in healthy subjects and detects small intra-individual changes. In addition, the QTcj calculated from uncompensated data was 17 ms shorter than that estimated from compensated data [32,33].  Fig. 5 presents an example from our database of analysis of QT dynamics in a healthy 36 year-old man using this method. The analysis was performed using Holter system Pathfinder incorporating Ventricular Dynamics Analyser sofware- “VERDA” (Del Mar Reynolds Medical, Hertford, UK) which utilises Neilson’s formula [32,33]. Pueyo et al [34] analysed the effect of QT/RR hysteresis using the database of the European Amiodarone Myocardial Infarction Trial (EMIAT). In this study, QT/RR hysteresis was characterized by two parameters: 1). QT lag based on QT interval dependence on preceding RR intervals, 2).characteristic adaptation profile i.e. the way in which QT reacts to RR changes. Analysis of QT adaptation lag revealed that the QT duration was influenced on the average of 140 seconds of preceding RR intervals. The influence of the most distant RR intervals was small in comparison to the most recent ones. Both strong dependence of QT on the preceding cardiac cycle, and individually variations of past heart rate contributed to the QT duration. QT dynamics parameters varied substantially between different patients, e.g. the QT lag values ranged from 3 to 215 seconds, and the way of adaptation ranged from 46 to 54 beats. Therefore, the authors concluded that QT/RR hysteresis pattern is highly individual and highlighted the necessity to use methods which takes into account the individual profiles [34].

Neilson’s method has been used in a few clinical studies. In patients with heart failure, the QT dynamics indices were significantly higher than in healthy volunteers, with mean reported values of J and QTcj of 0.38 and 461 ms, repectively.[35] QTcj and J values correlated positively with NYHA functional class [35]. The highest values of QT dynamics parameters (J of 0.8 and QTcj of 526 ms) have been described in patients with ischemic heart disease and cardiac arrest [36]. The method has also been used to examine drug-induced changes QT dynamics. For example, it was used in an evaluation of the effects of sildenafil citrate, a phosphodiesterase inhibitor, on J and QTcj [36]. At high plasma concentration it acts as a class III antiarrhythmic drug, with potential to prolong repolarisation and cause malignant ventricular arrhythmias [38]. In the contrary to previous study, sildenafil citrate had no adverse effects on QT dynamicity [37,38]. Several drugs have been withdrawn from the market because of documented prolongation of repolarisation [39,40]. QT dynamics parameters seems to be a promising new method to examine the effects of drugs on ventricular repolarisation [10,12,32,33].

QT dynamics is influenced by a variety of factors, such as oestrogens, electrolyte disturbances, the renin-angiotensin-system-aldosterone system, down-regulation of ion currents and autonomic nervous activity [35]. The effect of the latter is particularly important, both by modulating heart rate as well as by directly influencing action potential duration [41]. The highest values of QT dynamics have been observed in the early morning hours when sympathetic activity is highest [42]. It is well known that the incidence of many adverse cardiovascular events, including malignant ventricular arrhythmias and sudden cardiac death is also highest during the morning hours [43-46]. Therefore QT dynamics might represent an independent predictor of arrhythmic risk.

## Conclusions

In clinical practice, when the general trend of QT dynamics needs to be assessed, a simple method, such as linear regression analysis, is recommended. When a more precise evaluation is needed, for example in drug studies, to estimate the risk of sudden cardiac death, or to monitor the evolution of repolarisation abnormalities, methods that provide a QT lag compensation should be applied. Researchers need to be fully aware that all QT dynamics methods require very high quality recordings and accurate long-term ECG analyses, including special care when T wave morphology is abnormal [15,32,34]. Clinicians and researchers should also be aware that no single formula for heart rate correction of the QT interval is universally applicable [47].
